# Plakoglobin Reduces the *in vitro* Growth, Migration and Invasion of Ovarian Cancer Cells Expressing N-Cadherin and Mutant p53

**DOI:** 10.1371/journal.pone.0154323

**Published:** 2016-05-04

**Authors:** Mahsa Alaee, Ghazal Danesh, Manijeh Pasdar

**Affiliations:** Department of Oncology, University of Alberta, Edmonton, AB, T6G1Z2, Canada; Peking University Cancer Hospital & Institute, CHINA

## Abstract

Aberrant expression of cadherins and catenins plays pivotal roles in ovarian cancer development and progression. Plakoglobin (PG, γ-catenin) is a paralog of β-catenin with dual adhesive and signaling functions. While β-catenin has known oncogenic function, PG generally acts as a tumor/metastasis suppressor. We recently showed that PG interacted with p53 and that its growth/metastasis inhibitory function may be mediated by this interaction. Very little is known about the role of PG in ovarian cancer. Here, we investigated the *in vitro* tumor/metastasis suppressor effects of PG in ovarian cancer cell lines with mutant p53 expression and different cadherin profiles. We showed that the N-cadherin expressing and E-cadherin and PG deficient ES-2 cells were highly migratory and invasive, whereas OV-90 cells that express E-cadherin, PG and very little/no N-cadherin were not. Exogenous expression of PG or E-cadherin or N-cadherin knockdown in ES-2 cells (ES-2-E-cad, ES-2-PG and ES-2-shN-cad) significantly reduced their migration and invasion. Also, PG expression or N-cadherin knockdown significantly decreased ES-2 cells growth. Furthermore, PG interacted with both cadherins and with wild type and mutant p53 in normal ovarian and ES-2-PG cell lines, respectively.

## Introduction

Ovarian cancer (OVCA), the fifth most prevalent cancer in women is the leading cause of all female reproductive cancer deaths worldwide, with an overall five-year survival rate of ~ 45% [1]. The major form of OVCA is the epithelial ovarian cancer (EOC), which accounts for ~80% of all ovarian neoplasms [2]. EOCs are classified into type I and type II [3]. Type I tumors are genetically stable, slow growing, and have relatively good clinical outcome. However, the majority of OVCA are type II. Over 90% of these tumors harbor p53 mutations, are genetically unstable, highly aggressive and have poor clinical outcome [4–6]. *TP53* mutations are believed to be an early event during the development of type II tumors and contribute to both metastatic progression and chemoresistance [7–12]. p53 is a transcription factor and tumor suppressor that plays essential roles in regulating cell proliferation, survival, senescence, apoptosis and metabolism [13]. In response to stress, p53 activates DNA damage response, cell cycle arrest and cell death [14,15]. Different posttranslational modifications and protein-protein interactions regulate p53 stability and functions [16]. We have identified plakoglobin (PG, γ-catenin) as a novel interacting partner of both wild type (WT) and mutant p53 (mp53) [17,18].

Plakoglobin is a member of the Armadillo family of proteins and a paralog of β-catenin [19,20]. Unlike, β-catenin, which only associates with adherens junctions and possesses well-known oncogenic functions, PG is a tumor/metastasis suppressor protein and participates in the formation of both adherens junctions and desmosomes [19,21]. PG can confer growth/metastasis inhibitory effects via its interactions with cadherins and induction of contact inhibition of growth [19]. In addition, it can interact with a number of intracellular partners including transcription factors [17–19,22–27]. We have shown that PG interacts with p53 and its tumor/metastasis suppressor function may, at least partially, be mediated by this interaction [17,18].

A number of studies have suggested that the loss of cadherin-catenin complex and activation of β-catenin oncogenic function play pivotal roles in the local invasion of ovarian tumor cells and subsequent metastasis [28–31]. Furthermore, the loss of heterozygosity of the PG gene (JUP) has been reported in sporadic OVCAs [32]. However, very little is known about the role of PG in OVCAs. In this study, we assessed the potential tumor/metastasis suppressor functions of PG in OVCAs, using the normal ovarian cell line IOSE-364 and OVCA cell lines OV-90 (PG and E-cadherin positive, mp53 expressing), ES-2 (PG and E-cadherin negative, N-cadherin positive and mp53 expressing), ES-2-PG (ES-2 transfectants expressing PG), ES-2-E-cad (ES-2 transfectants expressing E-cadherin) and ES-2-shN-cad (ES-2 cells in which N-cadherin has been knocked down). We examined PG levels, localization and interactions with E- and N-cadherin and p53 and assessed the growth, migratory and invasive properties of various cell lines. The results showed that PG interacted with both cadherins and p53. Exogenous expression of E-cadherin or PG or knockdown of N-cadherin significantly reduced the migration and invasion of ES-2 cells. Furthermore, PG expression and N-cadherin knockdown but not E-cadherin expression significantly reduced ES-2 cells growth.

## Materials and Methods

### Cell lines and culture conditions

IOSE-364 (hereafter IOSE) were grown in a 1:1 M199 and MCDB M105 media plus 5% FBS and 1% PSK (Penicillin, Streptomycin, Kanamycin). OV90 cells were maintained in the same M199 and MCDB M105 media plus 15% FBS and 1% PSK. ES-2 cells were grown in McCoy’s 5a media completed with 10% FBS and 1% PSK. ES-2-E-cad and ES-2-PG cells were grown in ES-2 media containing 400 μg/ml (selection) or 200 μg/ml (maintenance) G418. ES-2-shNcad transfect ants were cultured in ES-2 media with 1μg/ml (selection) or 0.5 μg/ml (maintenance) puromycin.

### Transfection

Plasmids encoding E-cadherin and PG have been described [33, 34]. Cultures of ES-2 cells in 60 mm or 100 mm dishes were transfected at 50–75% confluency with 10–25μg of DNA using calcium phosphate. Twenty hours after transfection, cells were rinsed with PBS and allowed to recover for 24 hours in complete growth media. To select stable transfectants, 72 h after transfection, media containing 400 μg/ml G418 (ES-2- PG and ES-2- E-cad transfectants) were added to cells and resistant colonies selected for 3–4 weeks. Resistant clones were maintained in 200 μg/ml G418 and screened for PG and E-cadherin expression by immunofluorescence and immunoblotting assays.

### N-cadherin knockdown

Human N-cadherin lentiviral shRNA plasmid [35] was used to transfect Phoenix-AMPHO cells using calcium phosphate. Lentiviral particles collected at 48 and 72 hours post transfection were combined and filtered using a 0.45μm low-protein binding filter. Lentiviral particles were used to transduce ES-2 cells in the presence of 8μg/ml polyberene (Santa Cruz, Canada). Puromycin-resistant stable cell lines expressing the N-cadherin shRNAs (ES-2-shN-cad) were isolated and the N-cadherin levels assessed by immunoblot and immunofluorescence.

### Immunoblot Analysis

Confluent 100 mm culture plates were rinsed with cold PBS and solubalized in SDS sample buffer (10 mM Tris–HCl pH 6.8, 2% (w/v) SDS, 50 mM dithiothreitol, 2 mM EDTA, 0.5 mM PMSF, 1mM NaF, 1mM Na_3_VO_4_). Equal amounts of total cellular proteins were separated by SDS-PAGE and transferred onto nitrocellulose membranes (Biorad, Canada). The membranes were incubated in specific primary antibodies overnight at 4°^C^ followed by the appropriate secondary antibodies at room temperature (Table 1). Membranes were scanned using an Odyssey CLx infrared imaging system.

**Table 1:** Antibodies and their respective dilutions in specific assays.

		Assay			
Primary antibodies	Species	WB	IP	IF	Source
p53	Mouse	1:1000	1:100	-	Santa Cruz, sc-126
Plakoglobin	Mouse	1:1000	1:100	1:100	Translab, 610254
E-cadherin	Mouse	1:1000	-	1:100	Translab, 610404
N-cadherin	Mouse	1:1000	-	-	Santa Cruz, sc-59987
Cytokeratin (pan-keratin)	Mouse	1:1000	-	-	Sigma, C-2931
Vimentin	Mouse	1:1000	-	-	Sigma, V-6630
β-actin	Mouse	1:1000	-	-	Santa Cruz, sc-47778
**Secondary antibodies**					
Anti-mouse IgG, light chain	Goat	1:15000	-	-	Jackson Immuno Research, 115-625-174
Anti-rabbit IgG, light chain	Goat	1:15000	-	-	Jackson Immuno Research, 211-652-171
Alexa fluor 488	Mouse	-	-	1:2000	Molecular Probes, A11029
Alexa fluor 546	Rabbit	-	-	1:3000	Molecular Probes, A11035
Rhodamine	Rabbit	-	-	1:400	Boehringer Mannheim, 605107
Rhodamine	Rat	-	-	1:400	Sigma, T4280

### Immunofluorescence

Confluent cell cultures were established on glass coverslips and rinsed with cold PBS containing 1mM each of NaF, Na_3_VO_4_ and CaCl_2_. Cells were fixed with 3.7% formaldehyde for 20 minutes and extracted with CSK buffer (50mM NaCl, 300 mM Sucrose, 10 mM PIPES pH 6.8, 3 mM MgCl_2_, 0.5% Triton X-100, 1.2 mM PMSF, and 1 mg/ml DNase and RNase; [17]) for 10 minutes. Coverslips were blocked with 4.0% goat serum and 50mM NH_4_Cl_4_ in PBS containing 0.2% BSA for 1 hour. Coverslips were then incubated in the specific primary antibodies for 1 hour followed by the secondary antibodies for 30 minutes at concentrations indicated in Table 1. Nuclei were counterstained with DAPI (1:2000). Coverslips were mounted in elvanol containing 0.2% (w/v) paraphenylene diamine (PPD) and viewed using a 63x objective lens of a Zeiss confocal microscope.

### Immunoprecipitation

Cultures were grown to confluency in 100 mm dishes and rinsed with cold PBS containing 1mM NaF, Na_3_VO_4_ and CaCl_2_. Cells were extracted in 1ml of lysis buffer (50 mM Tris-HCl pH 7.5, 150mM NaCl, 1% NP-40, 0.5% sodium deoxycholate, 0.7μg/ml Pepstatin, 1 mM Na_3_VO_4_, 1 mM NaF, and protease inhibitor cocktail) for 20 minutes on a rocker at 4°C. Cells were scraped and centrifuged at 48000xg for 10 minutes. Supernatants were processed for immunoprecipitation with p53 and PG antibodies (Table 1) and 40 μl protein G agarose (Thermo Fisher Scientific, Canada) beads overnight on a rocker-rotator at 4°C. Samples were then centrifuged at 14000xg for 2 min, the beads were removed and the supernatants processed for a second immunoprecipation for 3 hours. Beads from the two immunoprecipitations were combined and washed three times with the lysis buffer. Immune complexes were solubilized in 60 μl SDS sample buffer, separated by SDS-PAGE and processed for immunoblot as described above.

### Growth, migration and invasion assay

For in vitro growth assay, 3x10^4^ cells from ES-2, ES-2-E-cad, ES-2-PG and ES-2-shN-cad cells were plated in a 24-well plate. At 1, 3, 5 and 7 days after plating, cultures were trypsinized and cells were counted. Each time point represents the average of three independent experiments.

For cell migration assays, 2×10^5^ cells were resuspended in 0.5 ml serum-free media and plated in the upper chamber of transwell inserts (3μm pore, 6.5mm diameter; BD Biosciences, CA, USA). Normal media containing 10% FBS was added to the lower chamber and cultures were incubated for 16 hours at 37°C. Inserts were then transferred into new dishes and rinsed with PBS to remove un-attached cells. Inserts were fixed with 3.7% formaldehyde (in PBS) for 2 minutes, permeabilized with 100% methanol for 20 minutes and stained with Giemsa stain for 15 minutes at room temperature. Following staining, membranes were viewed under an inverted microscope using a 20x objective lens and photographed.

For Matrigel invasion assays, cells were starved in serum-free media for 24 hours prior to the assay. For each cell line, 5×10^4^ cells in 0.2 ml serum-free media were plated in the top compartment of Matrigel-coated invasion chambers (8 μm pore PETE membrane; BD Biosciences). Fibroblast conditioned media (0.8 ml) was added to the bottom chambers and plates were incubated overnight at 37°C. After 16 hours, membranes were recovered and processed as described for the migration assay. Mounted membranes were viewed under a 20x objective lens of an inverted microscope and photographed.

The migrated/invaded cells were counted in 5 random fields for each membrane using ImageJ Cell Counter program. Numbers for each cell line were averaged and normalized to those of the normal cell line or parental untransfected cells and histograms constructed. Histograms represent the average of at least 3 independent assays for each cell line.

### Statistical analysis

Values are presented as means± SD. Statistical differences between groups were assessed by Student’s t-tests. *P*-value <0.05 was considered significant.

## Results

### Protein expression of epithelial and mesenchymal markers and p53 in various OVCA cell lines

**Fig 1:**
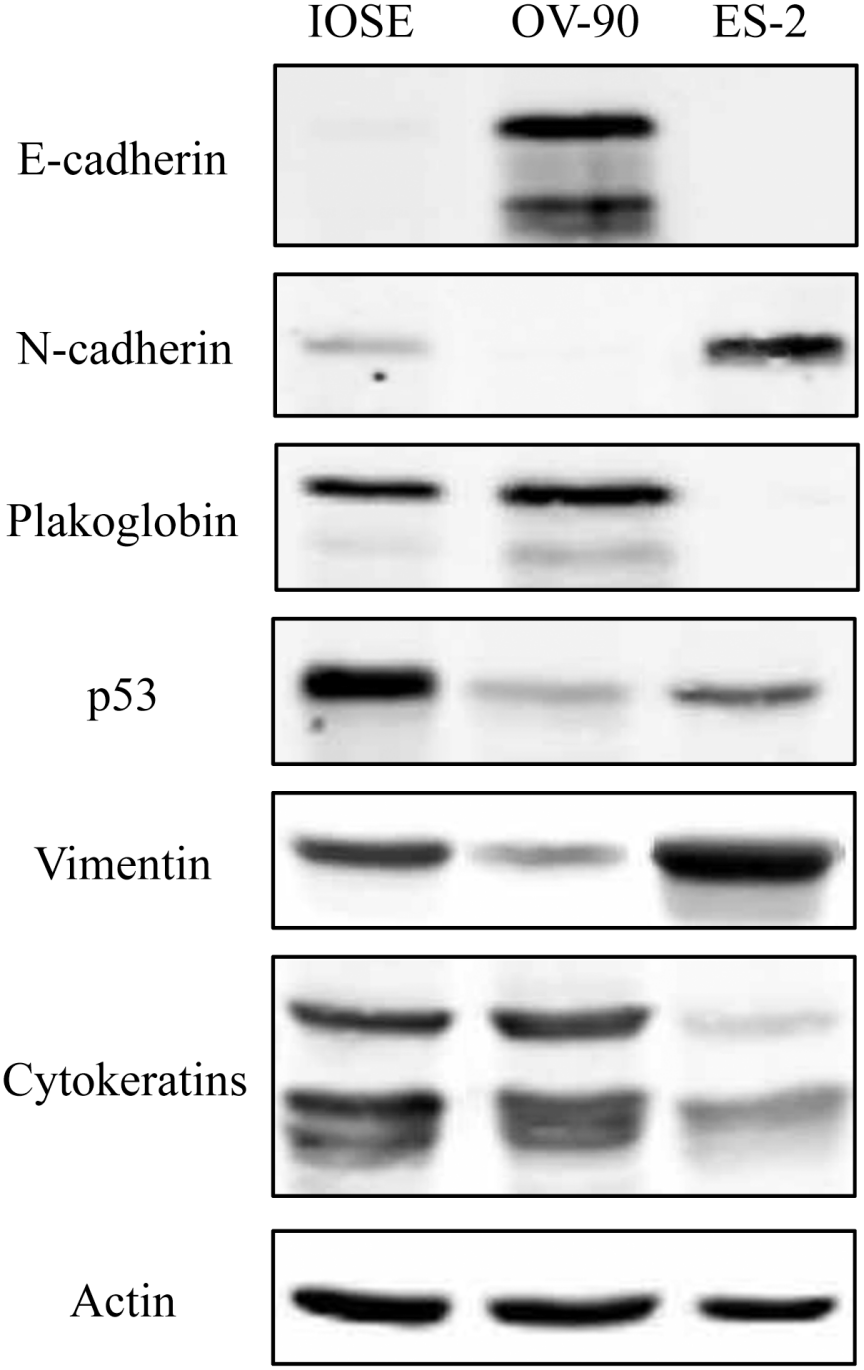
Protein expression of epithelial and mesenchymal markers and p53 in OVCA cell lines. Total cell lysates from IOSE-364, ES-2 and OV-90 cells were processed for immunoblot analysis using **N-cadherin**, **E-cadherin**, **plakoglobin**, **vimentin**, **cytokeratins** and **p53 antibodies**. Equal loadings were confirmed by processing the same lysates with actin antibodies.

Protein expression of E-cadherin, N-cadherin, plakoglobin, cytokeratins, vimentin and p53 in IOSE, ES-2 and OV-90 cells were detected using immunoblot analysis (Fig 1). IOSE cells had very little, if any, E-cadherin and expressed N-cadherin and PG. These cells also expressed cytokeratins, vimentin and p53. These observations were consistent with previous findings indicating that normal OSE cells displayed both epithelial and mesenchymal markers [36]. In contrast, OV-90 cells that express mp53 [37] had no detectable N-cadherin, low levels of vimentin and high levels of epithelial markers including E-cadherin, PG and cytokeratins. ES-2 cells, which also express mp53 [38, 39], displayed a more mesenchymal phenotype, lacked E-cadherin and PG and expressed N-cadherin, vimentin and very low levels of cytokeratins.

### Levels and localization of E-cadherin, N-cadherin and plakoglobin in normal and carcinoma ovarian cell lines

**Fig 2:**
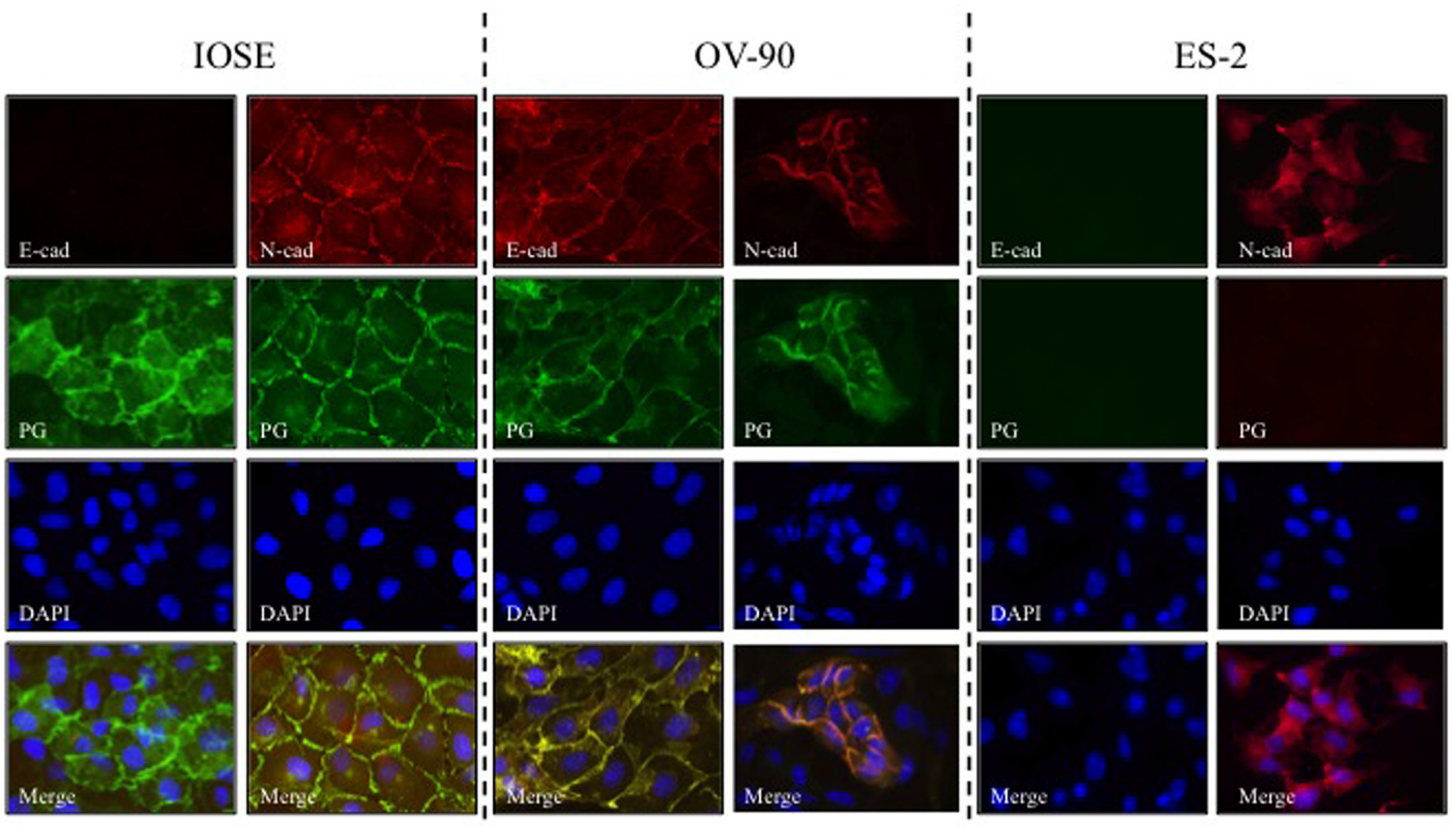
Levels and localization of E-cadherin, N-cadherin and plakoglobin in normal and carcinoma ovarian cell lines. IOSE-364, ES-2 and OV-90 cells were grown on coverslips and processed for double immunofluorescence staining. E-cadherin (**E-cad**, red) or N-cadherin (**N-cad**, red) and plakoglobin (**PG**, green) antibodies were used at concentrations indicated in Table 1. Nuclei were stained with DAPI (blue). Bar, 25 μm.

Subcellular distribution and potential co-localization of E-/N-cadherin with PG were examined by double immunofluorescence staining (Fig 2). In IOSE cells, consistent with the immunoblot results, E-cadherin levels were undetectable whereas N-cadherin and PG were expressed at high levels and were co-distributed at the membrane (Fig 2, **IOSE**). In OV-90 cells, high levels of E-cadherin and PG were present and were colocalized at the membrane. We also detected scarcely distributed small patches of N-cadherin positive cells in OV-90 cultures. In these patches, N-cadherin was colocalized with PG (Fig 2, **OV-90**). In ES-2 cells, there was no detectable E-cadherin or PG, whereas they expressed high levels of N-cadherin, which was distributed throughout the cytoplasm (Fig 2, **ES-2**). Consistent with the absence of PG and adhesive junctions, ES-2 cells exhibited significantly less cell-to-cell contact and their morphology was distinctly different than IOSE and OV-90 cells.

### The absence of E-cadherin and PG expression and the presence of N-cadherin contribute to the migratory and invasive properties of ES-2 cells

**Fig 3:**
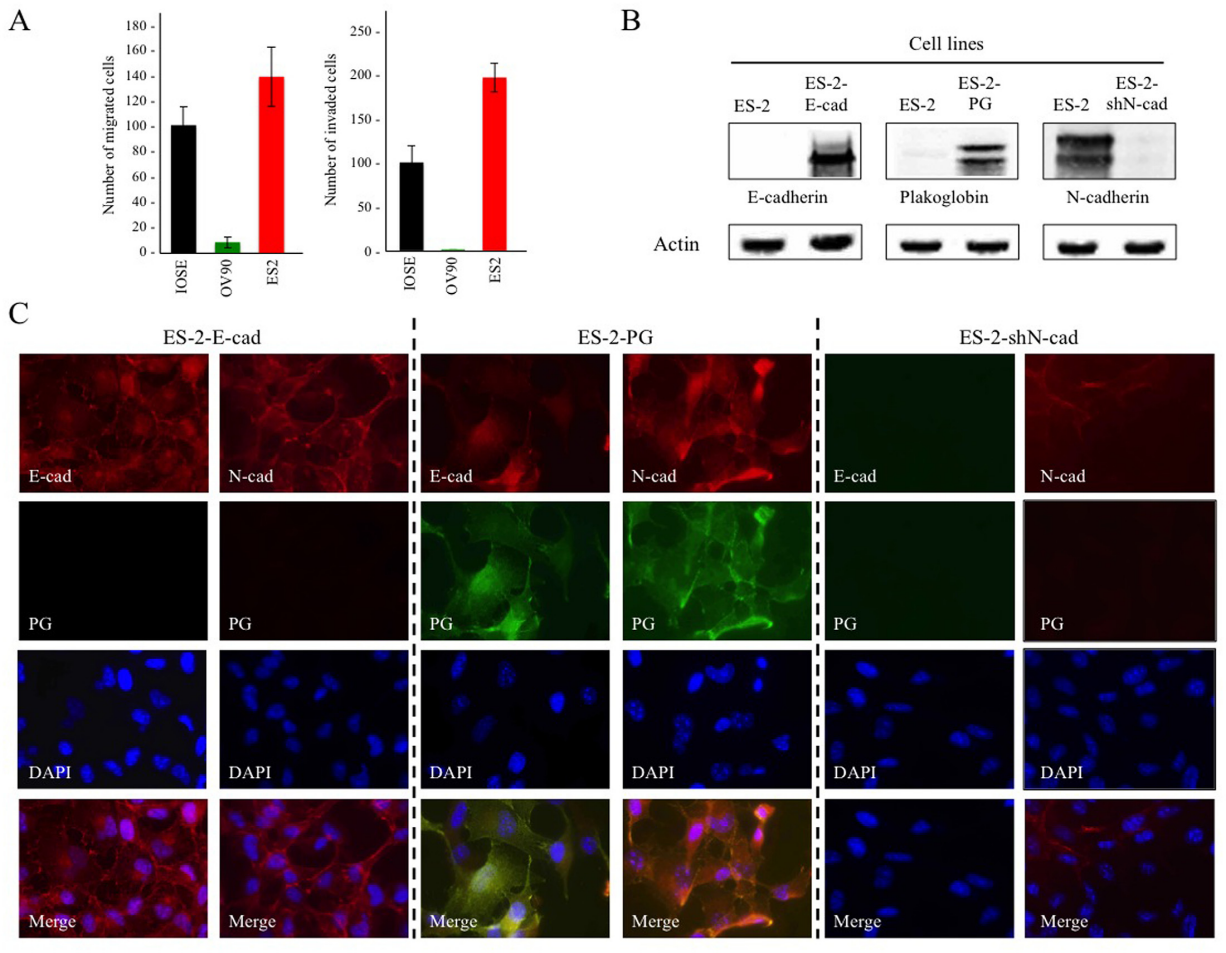
**(A) Migration (Left) and invasion (Right) of IOSE-364, OV-90, ES-2 cells.** Cultures were processed for in vitro migration and invasion assays as described in Materials and Methods. The number of migrated/invaded cells was normalized to those of the IOSE-364 cells. **(B) Expression of E-cadherin, N-cadherin and plakoglobin in ES-2 transfectants expressing E-cadherin (ES-2-E-cad) or plakoglobin (ES-2-PG) or N-cadherin shRNAs (ES-2-shN-cad).** Stable transfectants were processed for immunoblotting using E-cadherin, plakoglobin and N-cadherin antibodies. To confirm equal loadings, the same cell lysates were processed with actin antibodies. **(C) Subcellular distribution and colocalization of E-cadherin, N-cadherin and plakoglobin in ES-2- E-cad, ES-2-PG and ES-2-shN-cad transfectants.** Stable transfectants were established on coverslips and processed for double immunofluorescence with E-cadherin **(E-cad, red)** or N-cadherin (**N-cad**, red) and plakoglobin (**PG**, green) antibodies. Nuclei were stained with **DAPI** (blue). Bar, 25 μm.

Previously, we have shown that the expression of PG in PG-deficient carcinoma cells that lack E-cadherin and express N-cadherin decreases their *in vitro* growth, migration and invasion [33, 34]. To examine whether PG had similar effects in OVCA cells, we first examined the migration and invasion properties of IOSE, OV-90 and ES-2 cells. Then, we exogenously expressed E-cadherin or PG or knocked down N-cadherin in these cells and assessed changes in their growth, migration and invasion. As depicted in Fig 3A, OV-90 cells showed significantly lower migration and invasion relative to IOSE cells (8.4% and 0.4%, respectively). In contrast ES-2 cells were significantly more migratory and invasive compare to IOSE cells (138% and 196.4%, respectively).

Exogenous expression of E-cadherin and PG and stable knockdown of N-cadherin in ES-2 transfectants was confirmed using immunoblot (Fig 3B) and immunofluorescence analyses (Fig 3C). In ES-2-E-cad cells (Fig 3B and 3C, **ES-2-Ecad**), E-cadherin was expressed and mainly localized at the membrane although it was also detected in the cytoplasm of the transfectants. Interestingly, PG expression in ES-2-PG cells (Fig 3B and 3C, **ES2-PG**) led to the upregulation of endogenous E-cadherin. In these cells, the exogenously expressed PG colocalized with both N-cadherin and E-cadherin (Fig 3B and 3C, **ES2-PG**). N-cadherin knockdown reduced the levels of the endogenous N-cadherin (>90%). Staining of these cultures with N-cadherin antibodies detected occasional cells that were barely stained (Fig 3B and 3C, **ES2-shN-cad**).

**Fig 4:**
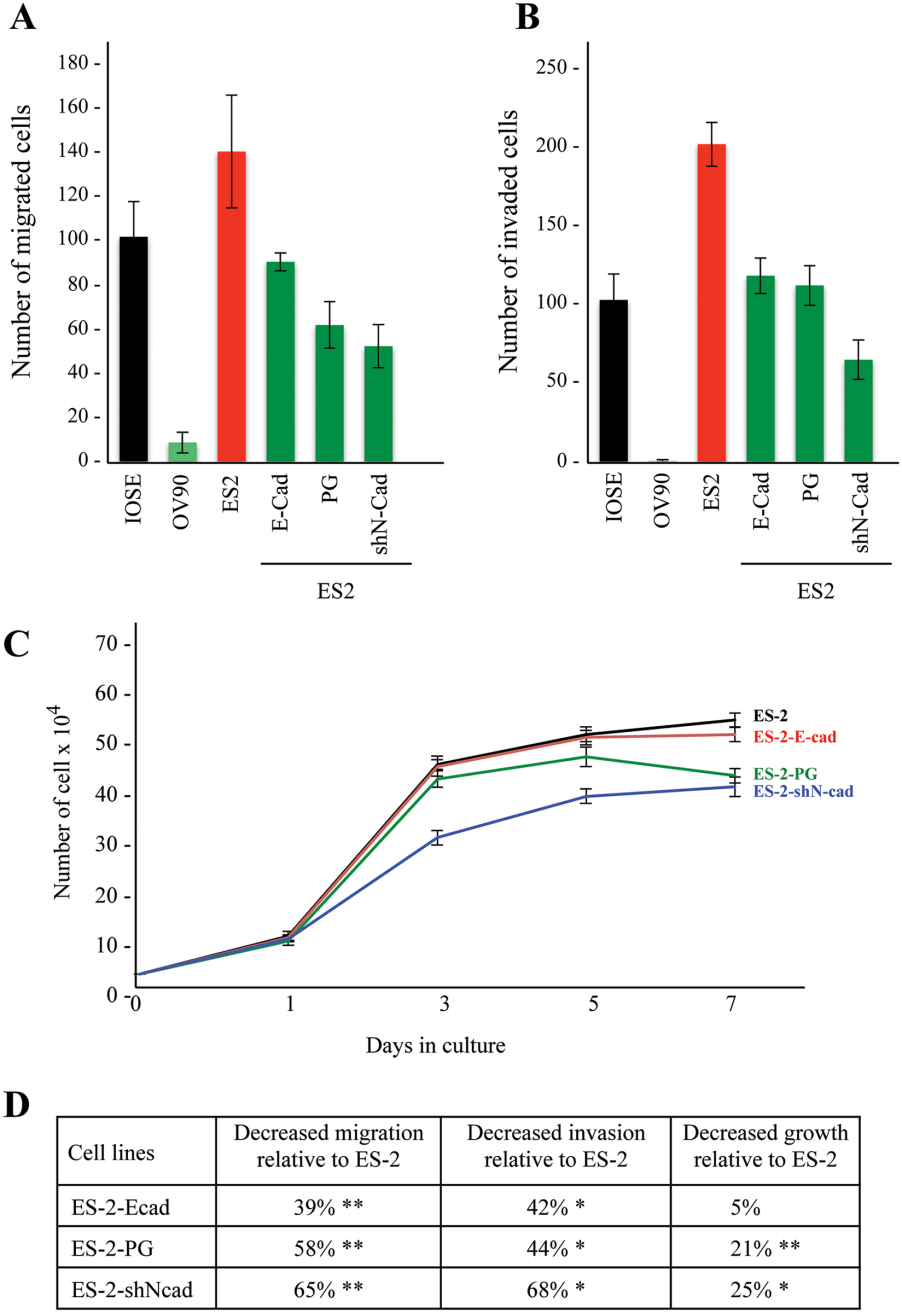
Migration, invasion and growth properties of normal and carcinoma ovarian cell lines. IOSE-364, OV-90, ES-2 and ES-2 transfectants (ES-2-E-cad, ES-2-PG, ES-2-shN-cad) were processed for **migration** (**A**) and **invasion (B)** assays as described in Materials and Methods. The number of migrated/invaded cells was normalized to those of the IOSE-364 cells. **(C)** Replicate cultures of ES-2 cells and ES-2 transfectants (E-cad, PG and shN-cad) were plated at single cell (3x10^4^) density. Cultures were counted at day 1, 3, 5 and 7. Each time point is the average of three independent experiments. **(D)** Summary of changes in growth, migration and invasion of ES-2 transfectants. Transfectants values were normalized to ES-2 cells. *p* values, * <0.05, ** < 0.001.

Assessment of the migration and invasion of ES-2 transfectants showed a significant reduction in both migration and invasion of ES-2-Ecad and ES-2-PG cells relative to parental ES-2 cells (Fig 4A, 4B and 4D). E-cadherin expression in ES-2 cells reduced migration and invasion of these cells by 39% and 42%, respectively. PG expression in ES-2 cells decreased migration and invasion by 58% and 44%, respectively. The effect of N-cadherin knockdown on migration was similar to that of PG expression, i.e. a reduction of 65% whereas the invasion of ES-2-shN-cad cells was significantly less than that of ES-2-E-cad and ES-2-PG cells (68% reduction) (Fig 4A, 4B and 4D).

We also compared the growth of ES-2 cells with those of ES-2-E-cad, ES-2-PG and ES-2-shN-cad transfectants (Fig 4C and 4D). At day 7, ES-2-E-cad cells showed similar growth rate to ES-2 cells while ES-2-PG and ES-2-shN-cad cells showed significantly lower growth than ES-2 cells (21% and 25% reduction, respectively, (Fig 4C and 4D). However, while ES-2-shN-cad cells showed decreased growth throughout the 7 days, ES-2-PG cultures showed decreased cell number after day 5, likely due to the induction of contact inhibition upon culture confluency (Fig 4C).

Taken together, these results suggested that expression of E-cadherin or PG or knockdown of N-cadherin effectively reduced migration and invasion of ES-2 cells. However, only PG expression or N-cadherin knockdown significantly decreased the growth of these cells.

### Interaction of plakoglobin and p53 in normal and ovarian carcinoma cell lines

**Fig 5:**
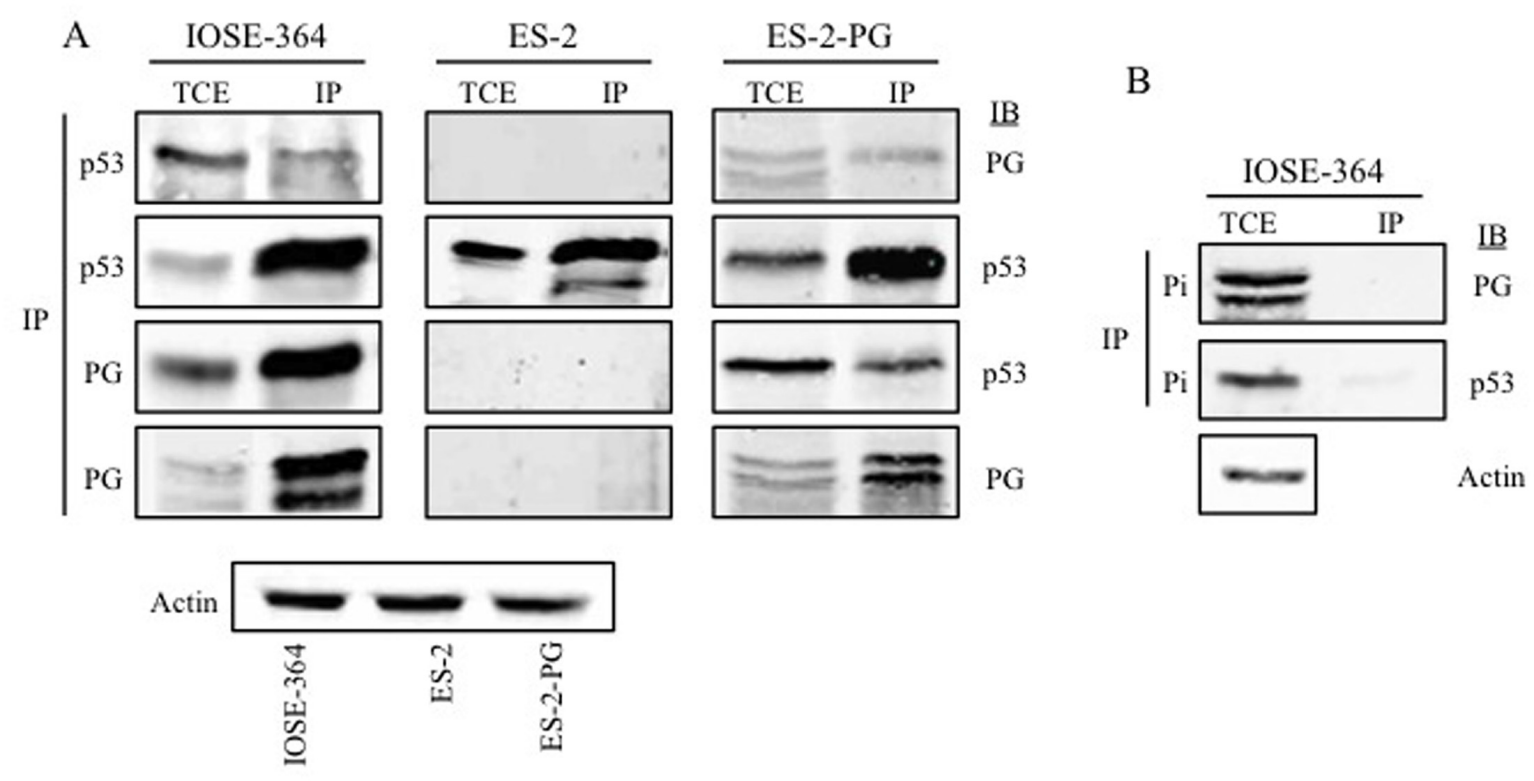
Interaction of plakoglobin and p53 in normal and ovarian carcinoma cell lines. Equal amounts of total cell extracts (TCE) from IOSE-364, ES-2 and ES-2-PG cells were processed for reciprocal and sequential immunoprecipitation (IP) and immunoblotting (IB) using p53 and plakoglobin antibodies **(A)** or preimmune antibodies **(B)** as described in Materials and Methods. The same lysates were processed with actin antibodies to confirm equal loadings. PG, plakoglobin; Pi, pre-immune.

We have shown that PG interacted with both WT and mp53 in various carcinoma cell lines and they both associated with promoters of a number of p53 target genes [17, 18, 40]. PG’s interactions with mp53 expressing carcinoma cells led to decreased growth, migration and invasion of these cells. To this end, we examined whether PG associated with p53 in OVCA cells. Total cell extract of IOSE, ES-2 and ES-2-PG cells were processed for reciprocal coimmunoprecipitation (co-IP) and immunoblotting with PG and p53 antibodies (Table 1). In IOSE cells PG antibodies coprecipitated p53 and PG (Fig 5). The reciprocal co-IP using p53 antibodies coprecipitated PG, further validating the interaction between PG and p53 in these cells. In ES-2 cells expressing exogenous PG and endogenous mp53, PG antibodies coprecipitated p53 and PG. In the reciprocal co-IP of ES-2-PG cells, p53 antibodies brought down both PG and p53 (Fig 5). In contrast, in ES-2 cells with no PG expression, p53 antibodies precipitated p53 only (Fig 5). Control immunoprecipitations with p53 and PG preimmune antibodies did not detect either protein in the total cell lysates (Fig 5B).

## Discussion

In the current study, for the first time, we investigated the *in vitro* tumor/metastasis suppressor effects of PG in EOC cell lines with mp53 expression and different cadherin profiles. We showed that ES-2 cells that express N-cadherin and are deficient in E-cadherin and PG were highly migratory and invasive. In contrast, OV-90 cells that express both E-cadherin and PG and very little N-cadherin were not migratory or invasive. The exogenous expression of PG or E-cadherin or knockdown of N-cadherin in ES-2 cells significantly reduced their migration and invasion. Our data showed that PG colocalized with both E-cadherin and N-cadherin in adhesion complexes. Consistent with these observations, we detected significant reduction in ES-2-PG and ES-2-shN-cad growth relative to ES-2 and ES-2-E-cad cells. Furthermore, PG interacted with WT p53 in IOSE cells and mp53 in ES-2-PG transfectants.

Cadherin switching from E- to N-cadherin is a critical step in the epithelial to mesenchymal transition (EMT)-mediated malignancies [41, 42]. EMT leads to the cell-cell junction disassembly, loss of cell polarity and gain of migratory and invasive properties [30, 43, 44]. While E-cadherin is an epithelial marker and a known tumor suppressor, N-cadherin is a mesenchymal marker and its expression is associated with a more migratory and invasive phenotype [44, 45]. Normal ovarian surface epithelial (OSE) cells express a combination of epithelial and mesenchymal markers. These cells do not have E-cadherin but express N-cadherin, catenins, vimentin and cytokeratins [36, 46–50]. In agreement with these reports, IOSE cells expressed N-cadherin and vimentin as well as catenins including PG, and cytokeratins. The exact role of E-/N-cadherin switch in the initiation and progression of ovarian carcinomas is not very clear since both cadherins can be expressed in ovarian tumors of different origins and at different stages [51, 52]. However, while a few studies suggest that E-cadherin is upregulated in OVCA effusions [52, 53], the great majority suggest that the loss or reduced levels of E-cadherin contribute to the transition from benign to borderline ovarian lesions, to poorly differentiated ovarian tumors, and to the local invasion and metastasis [28, 47, 54–58]. Consistent with the tumor suppressor activities of E-cadherin, downregulation/lack of E-cadherin expression due to the high levels of its transcriptional repressors Snail, Twist and ZEB-2 has been associated with the migratory and invasive properties of ES-2 and other OVCA cells [59–71]. In addition, E-cadherin suppresses growth and metastasis via inhibiting receptor tyrosine kinase signaling and PI3k/Akt pathways [72, 73]. In agreement with these studies, we showed that ES-2-E-cad cells had significantly lower migration and invasion (39% and 42%, respectively) compared to ES-2 cells.

Although N-cadherin is expressed in normal OSE, its expression is generally associated with increased migration and invasion of OVCA [74–76]. N-cadherin levels have been shown to be elevated in cell lines expressing Snail and ZEB-1, as well as, in patients with higher FIGO tumor grade and metastasis [51, 64, 77]. Exogenous expression of MUC4 in SKOV3 cells led to the downregulation of E-cadherin, upregulation of N-cadherin and increased motility. N-cadherin knockdown in these cells reduced MUC4 induced motility, concurrent with decreased activity of ERK1/2, AKT and MMP9 [78]. Supporting these studies, a selective anti-N-cadherin antibody (Exherin, ADH-1) was recently shown to be effective in stabilizing disease progression in two OVCA patients in a small phase I clinical study, which assessed patients with various solid tumors [79]. Here, we showed that relative to ES-2 cells, the migration and invasion of ES-2-shN-cad transfectants were reduced by 65% and 68%, respectively. Furthermore, knocking down N-cadherin was much more effective in reducing migration and invasion than expressing E-cadherin in ES-2 cells. Similarly, while E-cadherin expression had very little effect (5%) in decreasing growth, PG expression or N-cadherin knockdown significantly reduced ES-2 cells growth (20%, 25%, respectively).

Unlike cadherins, very little is known about the role of PG in OVCA. PG has been shown to have growth/metastasis inhibitory function, both *in vitro* and *in vivo* [19]. This function of PG can be mediated by stabilizing /sequestering N-cadherin and induction of contact inhibition of growth and/or interacting with different cellular proteins including transcription factors [17–19, 27,34,40,80–82]. Here, the exogenous expression of PG significantly reduced migration and invasion of ES-2 cells (58% and 44% respectively). The effect of PG on inhibiting migration was significantly higher than that of E-cadherin. Since PG expression is necessary for the formation of both adherens junctions and desmosomes [19,33], this may suggest that PG reduced migration via association with N-cadherin and formation of junctions as well as interaction with transcription factors and regulation of gene expression. Interaction of PG with several transcription factors such as TCF/LEF, CBP, SOX4 and p53 has been reported previously [17, 22–26, 81]. We have shown that PG interacted with mp53 in several carcinoma cell lines and they both associated with promoters of a number of p53 target genes including tumor suppressors *SFN* (14-3-3s) and *NME1*and the oncogenic genome organizer *SATB1*. Furthermore, these associations were concurrent with reduced growth, migration and invasion [17,18]. PG also regulated the expression of HAI-1 and reduced migration in a p53 dependent manner in NSCLC cells [27]. Here, we showed that PG interacted with WT p53 in IOSE cells and with mp53 in ES-2-PG cells. p53 regulates the expression of EMT markers such as Twist, Snail and Slug [82–86]. We detected low levels of E-cadherin in ES-2-PG cells upon PG expression. Whether this E-cadherin expression is due to the downregulation of E-cadherin transcriptional repressors via PG-p53 interaction or stabilization of E-cadherin protein via its interaction with PG warrants further studies.

In summary, this is the first demonstration of the role of PG in OVCA cells. Our data showed that exogenous expression of PG or knockdown of N-cadherin were more effective than expression of E-cadherin in inhibiting the growth, migratory and invasive properties of ES-2 cells. These results suggest that PG expression sequestered tumor/metastasis promoting activities of N-cadherin. Induction of E-cadherin expression in ES-2 cells expressing exogenous PG, which interacted with the endogenous mp53, raises the possibility that PG may also be involved in the regulation of p53 target genes involved in migration and invasion. Collectively, the results suggest that PG may act as a tumor/metastasis suppressor in OVCA, as has been shown for other cancers. The larger implication of our studies is the potential of PG as a therapeutic target for the majority of OVCAs with mp53 and N-cadherin expression.
